
JOURNAL INFORMATION
==============================
NLM Title Abbreviation: ScientificWorldJournal
Journal ID: ScientificWorldJournal
NLM Journal ID: 101131163
Journal ID: TSWJ

The Scientific World Journal

ISSN: 2356-6140
EISSN: 1537-744X
Publisher: Wiley

ARTICLE INFORMATION
==============================
PMCID: PMC6084143
PMID: 30147547
DOI: 10.1100/tsw.2001.249
Article ID: 354636
Article version: 1
Subjects: Short Report

Processivity of C-Myc-Induced Telomerase Activation in Rat Cell Lines

Sasgary Soleman
Schmidt Gerlinde
Skrzypek Barbara
Cerni Christa *
Institute of Cancer Research, University of Vienna, A-1090, Austria
*Corresponding author.
Publication date: 2001
Electronic publication date: 2001 Nov 2
Volume: 1
First page: 71
Last page: 71
Copyright: Copyright © 2001 Soleman Sasgary et al.
Copyright year: 2001
License: This is an open access article distributed under the Creative Commons Attribution License, which permits unrestricted use, distribution, and reproduction in any medium, provided the original work is properly cited.
License URL: https://creativecommons.org/licenses/by/3.0/

==============================
Miami *Nature Biotechnology* Short Reports
*TheScientificWorld* (2001) 1 (S3), 71SR
ISSN 1532-2246; DOI 10.1100/tsw.2001.249

### PROCESSIVITY OF C-MYC-INDUCED TELOMERASE ACTIVATION IN RAT CELL LINES

Soleman Sasgary, Gerlinde Schmidt, Barbara Skrzypek, and Christa Cerni*

Institute of Cancer Research, University of Vienna, A-1090 Austria
* Corresponding author.

**INTRODUCTION.** It is suggested that reactivation of telomerase in cancer cells is a prerequisite for tumorigenicity (1), at least for human cells where initial telomere lengths of approximately 10 kb limit extensive proliferation (2). Recently, c-myc was found to induce expression of the catalytic component of telomerase, TERT, in human cell strains (3). Since c-myc expression is physiologically induced during G1/S-phase progression and found deregulated in a variety of malignant tumors, the question arose to what extent overexpression of c-myc oncogene might contribute to telomere elongation. We created transformed rat cell lines with inducible c-myc- or c-H-ras-oncogene, respectively, and analyzed the effect of c-myc expression on telomere lengths.

**METHODS.** Primary Fisher rat embryo cells (REC) were transfected with various c-myc- and c-H-ras-constructs. Individual transformed clones were isolated and further cultivated with or without myc and ras- induction and analyzed for gene and protein expression (Northern- and Western-blot analysis), telomerase activity (TRAP assay) and telomere lengths (Pulsed Field Gel Electrophoresis).

**RESULTS.** Normal REC have low telomerase activity and telomeres up to 100 kb. REC-derived transformed cell lines expressing c-myc and c-H-ras showed high levels of telomerase activity comparable to that found in human HeLa cells (Fig.1). which in turn induced significant telomere lengthening (Fig. 1, compare lane 1 and 3). Individual high-molecular-weight telomere bands were used to calculate the gain of telomeric sequences during passaging. We found that c-myc-activated telomerase added up to several hundred nucleotides per cell generation. Conversely, inactivation of c-mycER$^{TM}$ by omitting Tamoxifen (TM) from the medium or reduction of c-myc levels indirectly by shutting off ras (4) was accompanied by morphological cell alteration, loss of telomerase activity (Fig. 2) and shortening of telomeres of up to 3000 nucleotides per cell generation.

**FIG. 1** **FIG. 2**

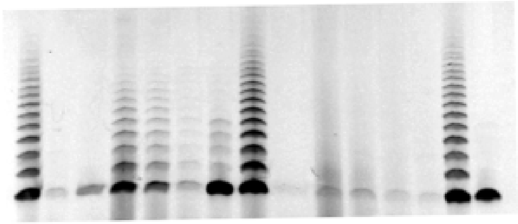

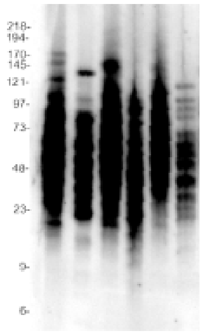

| lane | 1 | 2 | 3 | 4 | 5 | 6 | 7 | 8 | 9 | 10 | 11 | 12 | 13 | 14 | 15 |
|---|---|---|---|---|---|---|---|---|---|---|---|---|---|---|---|
| MR-13 | - | - | | | | | | | | | | | | | - |
| HeLa | + | + | | | | | | + | | | | | | + | - |
| RNase | | + | + | | | | | | + | | | | | | - |

| lane | 1 | 2 | 3 | 4 | 5 | 6 |
|---|---|---|---|---|---|---|
| transfer | 13 | 12 | 30 | 29 | 15 | 15 |
| Dex | + | - | + | - | + | - |

**CONCLUSION.** In rat cells, telomerase activity can be induced by overexpression of c-myc, which was achieved in REC clones only in presence of c-H-ras oncogene. C-myc-mediated activation of telomerase induced a substantial elongation of telomeres. The extent of both, telomerase activation and telomere elongation depending on c-Myc levels. Conversely, reduction of c-Myc caused a dramatic loss of telomeres which exceeded by far the normal loss of telomere sequences due to the end replication problem of DNA synthesis. These data suggest that c-myc contributes to telomere elongation or maintenance not only by acting on E-box sequences in the TERT promoter, but moreover by activating other genes involved in the proper structure of chromosomal ends.

**REFERENCES.**

1. Hahn, W.C., Counter, C.M., Lundberg, A.S., Beijersbergen, R.L., Brooks, M.W., and Weinberg, R.A. (1999) Nature 400, 464
2. Bryan, T.M. and Cech, T.R. (1999) Curr. Opin. Cell Biol. 11, 318
3. Wang, J., Xie, L.Y., Allan, S., Beach, D., and Hannon, G.J. (1998) Genes Dev. 12, 1769
4. Sears, R., Leone, G., DeGregori, J., and Nevins, J.R. (1999) Mol. Cell 3, 169

Supported by the Herzfelder'sche Familienstiftung.
